# Determining the distribution loss of brown eared-pheasant (*Crossoptilon mantchuricum*) using historical data and potential distribution estimates

**DOI:** 10.7717/peerj.2556

**Published:** 2016-10-19

**Authors:** Yilin Li, Xinhai Li, Zitan Song, Changqing Ding

**Affiliations:** 1 School of Nature Conservation, Beijing Forestry University, Beijing, China; 2 Forensic Center of Wildlife, Nanjing Forest Police College, Nanjing, China; 3 Key Laboratory of the Zoological Systematics and Evolution, Institute of Zoology, Chinese Academy of Sciences, Beijing, China

**Keywords:** Geographic information system, MaxEnt, Forest cover, Historical data, Brown eared-pheasant

## Abstract

We analyzed the synchronous relationship between forest cover and species distribution to explain the contraction in the distribution range of the brown eared-pheasant (*Crossoptilon mantchuricum*) in China. Historical resources can provide effective records for reconstructing long-term distribution dynamics. The brown eared-pheasant’s historical distribution from 25 to 1947 CE, which included the three provinces of Shaanxi, Shanxi, and Hebei based on this species’ habitat selection criteria, the history of the forests, ancient climate change records, and fossil data. The current species distribution covers Shaanxi, Shanxi, and Hebei provinces, as well as Beijing city, while Shanxi remains the center of the distribution area. MaxEnt model indicated that the suitable conditions of the brown eared-pheasant had retreated to the western regions of Shanxi and that the historical distribution area had reduced synchronously with the disappearance of local forest cover in Shanxi. We built a correlative relationship between the presence/absence of brown eared-pheasants and forest coverage and found that forest coverage in the north, northeast, central, and southeast areas of the Shanxi province were all less than 10% in 1911. Wild brown eared-pheasants are stable in the Luliang Mountains, where forest coverage reached 13.2% in 2000. Consequently, we concluded that the distribution of this species is primarily determined by vegetation conditions and that forest cover was the most significant determining factor.

## Introduction

The issue of species distribution is of fundamental interest to ecologists (Guisan & Thuiller, 2005). Understanding the biogeographic and ecological characteristics of species distribution decline is a key area of research in conservation science. Long-term historical data could offer insight into understanding the ecological and biogeographic characteristics of species distribution (Chapron et al., 2014; Rondinini & Visconti, 2015), and develop our understanding of long-term species distribution change (Yang et al., 2016). In particular, accurate information on patterns and drivers of distribution change under different environmental conditions is fundamental for developing appropriate management strategies for threatened species (Turvey, Crees & Di Fonzo, 2015).

A basic understanding of species distribution is necessary to design conservation strategies in future. Since the first studies using species distribution models (SDMs) appeared in the 1980s, the number of published studies using SDMs has increased exponentially (Lobo, Jiménez-Valverde & Real, 2008). The aim of these models is to geographically represent the ecological niche of a target species and to evaluate these models to represent potential real world distributions (Soberón & Nakamura, 2009). Due to the limited locational information available for determining species distribution areas and the restricted number of environmental variables used in the study of historical distributions, some diverse algorithms were successfully for model historical biogeographic information, such as MaxEnt, DesktopGarp, and Open-Modeller (Stigall Rode & Lieberman, 2005; Zhang et al., 2013). However, current studies address time-scales of less than 10 years (Davies, Colombo & Hanley, 2014), some researches over periods more than 20 years (Turvey, Crees & Di Fonzo, 2015). Therefore, an increasing awareness of the need to integrate historical datasets into conservation research and environmental management (Turvey, Crees & Di Fonzo, 2015).

Some ecosystems in China have also experienced escalating natural resource over exploitation and habitat modification for several millennia, and these long-term impacts are likely to have substantially shaped the composition and distribution of regional faunas before the recent historical era (Elvin, 2004; Ren, 2007). Environmental variables, such as the percentage of vegetation cover and characteristics of the vegetation can exert direct or indirect effects on a species (Austin, 2002). Vegetation is one of the most widely used indirect indicators of the distribution of terrestrial animal species (Austin, 1991). It is likely that historical changes to vegetation may influence species distributions and reduce the geographic ranges of animals, leading to smaller, isolated species groups. This can result in a higher risk of extinction. Therefore, understanding how species respond to vegetation changes is crucial for helping conservation managers identify and implement appropriate management strategies given these vegetation changes.

In this study, we focus on the brown eared-pheasant (*Crossoptilon mantchuricum*) due to its unique characteristics, described as follows. As an endangered Phasianidae bird endemic to China (Liu, Su & Ren, 1991), the brown eared-pheasant has been listed as a vulnerable (VU) globally threatened species by the International Union for Conservation of Nature (IUCN) as a result of population decline and habitat fragmentation (International Union for Conservation of Nature, 2015). Among endangered species, this pheasant has one of the highest priorities for conservation in China base on small population and habitat fragmentation (CITES, 2015; Zheng & Wang, 1998). This species is a montane bird and is sensitive to climate variation (Li, Tian & Li, 2010). It lives in coniferous and mixed coniferous-broadleaf forests, where it roosts in tall trees at different elevations in different seasons, and breeds in coniferous forests at 2,000 m elevation (Liu, Su & Ren, 1991). This bird is active only between the 800 m and 2,600 m elevation range (Pang, Ma & Liu, 2009; Xu et al., 1998). It has a well-known biology and life history. Its distribution range has historically been wide and continuous (Wang, Chen & Lai, 1985). Its current range includes three discontinuous distribution areas (the Shaanxi, Shanxi, and Hebei provinces, including Beijing) (Li & Wei, 1993; Zhang, Zhang & Song, 2000). This historically broad distribution range has declined dramatically in the Shanxi province (Liu, Su & Ren, 1991). Meanwhile, forest coverage in this region has declined sharply (Qu & Mi, 2009a).

In this study, we aimed to (i) analyze the relationship between regional forest cover and the historical distribution of the brown eared-pheasant and (ii) understand its distribution in response to changes in forest cover. Understanding the long-term dynamics of the distributional changes would be helpful to offer insight into the mechanism behind the decline and endangerment of the species and improve conservation strategies (Yang et al., 2016).

## Materials and Methods

### Sources of historical distribution data

From the early Western Han Dynasty (156 BC) to the late Qing Dynasty (1911 CE), soldiers decorated their hats using the tail feathers of the brown eared-pheasant as symbols of magnificence and might (Zheng, 1978). Brown eared-pheasant were offered as tributes to kings in the Tang (618–907 CE) and the Song (960–1127 CE) Dynasties. Therefore, regions where brown eared-pheasants were found, together with the corresponding date, were recorded in ancient books and local archives. The information of species distribution in gazetteers having systematic compilation and local ecological data can be considered as an important complementary for historical distribution reconstruction (Yang et al., 2016). These gazetteers provided the historical distribution data and time data used in this study.

Historical distribution data and dates were obtained from literatures (51 ancient books, 149 references, and 7 monographs). Such data are becoming increasingly available from databases, web sites, and museum collections, providing a rich empirical basis for making predictive maps (Crawford & Hoagland, 2009). We aggregated all of the categories of historical distribution data to identify the total historical distribution range of this species in China from 25 to 1947 CE. This was a reasonable approach because the species is mostly sedentary, with high-site fidelity and limited dispersal ability, features that prevent full re-colonization.

The names and ranges of some counties in the historical literature have changed over time. We avoided errors by collating records of changes from county annals to obtain their present names. Longitude and latitude were considered to reveal geographical trends in species distribution (Real et al., 2003). The spatial analysis “function-identify” of ArcGIS was used to extract the longitude and latitude of the centroids of the modern counties as the historical counties location (Zhang et al., 2013) on the basis of China’s county-level administrative map (http://www.webmap.cn/). Filters are essential for further analysis, because historical records may contain potential errors or uncertainties (Yang et al., 2016). First analyze the habitat, morphological characteristics, and life habits of the brown eared-pheasant in written descriptions, then consider the changes in vegetation and climate in these counties’ records base on thousands of years in Chinese history, and combining suitable habitat characteristics of the brown eared-pheasant, finally we judged the authenticity of 56 historical records.

Some historical records were filtered. Here are the major ones: Fossils pertaining to brown eared-pheasant have been found in Zhoukoudian in Beijing, although they are very rare (Tsen-Hwang, 1935). Pleistocene fossils of *Crossoptilon jiai sp. Nov* were found in Zhoukoudian (Hou, 1982). At the same site, the fossil belt (Early Pleistocene) and occipitalia fossils (Late Pleistocene) were suspected to belong to *Crossoptilon harmani* (Data Source: National Infrastructure of Mineral Rock and Fossil Specimen Resources) (Hou, 1993). *Crossoptilon harmani* may be a primitive species (Tsan et al., 2003; Wu et al., 2005), and has a distant genetic relationship with the brown eared-pheasant (Shi, Zhang & Liu, 2001; Li, Huang & Lei, 2015). Therefore, we did not consider Zhoukoudian Beijing as the historical distribution of the brown eared-pheasant in this study.

Evolution of the brown eared-pheasant occurred in the Pliocene (Li, Huang & Lei, 2015), with adults adapting to resist temperatures of −42 °C (Liu, Su & Ren, 1991). Climate change resulted in drought at the end of the Early Pleistocene in northeast China (Zeng et al., 2011). The climate contrasted between the ice age and an interglacial period (Sosdian & Rosenthal, 2009). The distribution of panda retracted during the Pleistocene glaciation in the Zhoukoudian area of Beijing (Zhang et al., 2013). In Liaoning in the Pleistocene interglacial, the climate was subtropical and tropical, with evergreen broad-leaved and evergreen deciduous broad-leaved forests (Dong, 2011). The climate fluctuations co-occurred with widespread local species extinction (Hugall et al., 2002). The environment was not suitable for the brown eared-pheasant in this region because of its history of climate and vegetation changes in northeast China. Furthermore, brown eared-pheasants had never been recorded in northeast China (Zheng, 1978). We did not consider Liaoning (Tieling, Shenyang, and Gaizhou) and Heilongjiang (Heihe) as the historic distribution range of this species.

Huating is located in the southeast of the Liupan Mountains in Gansu and has only one record of brown eared-pheasant. Fengxiang and Longxian in Shaanxi are adjacent to Huating county in Gansu province. These sites are isolated and do not share a consecutive distribution with other locations that have historical records of brown eared-pheasant, but they are close to the distribution area of the Blue eared pheasant. Therefore, we considered that the three records from these locations were likely to pertain to blue eared pheasant.

### Sources of current distribution data

The geographical coordinates and time data referring to modern occurrences (1948–2000) were obtained from the Site Record Database for Chinese Galliformes, which includes extensive distributional data. The distribution data were collected from Chinese bird database, monographic research, journals, and other literature sources, ornithology monographs published locally and abroad, and specimens in collections at scientific research institutes and universities, all of which were confirmed by expert evaluation (Zhang & Ding, 2007). 45 occurrence positions were uploaded into a geographic information system (ArcGIS 10.0), and overlaid with layers representing elevation (http://www.resdc.cn) and the borders of administrative regions. We checked the longitude and latitude of sites with 45 modern occurrences using ArcGIS10.0 (Xi’an 1980 Geographic Coordinate System).

The historical and current distribution for the brown eared-pheasant in China were then reconstructed by integrating historical and current records within ArcGIS10.0. Using the number and abundance of data, it is possible to reconstruct the distribution change of the brown eared-pheasant.

### Distribution comparison

GIS are powerful tools for studying the geographical distribution of species, and they are widely used in the management of nature reserves. We used ArcGIS10.0 to analyze the historical and current distribution records and investigate the reasons for the reduction of the geographical distribution of the brown eared-pheasant.

The brown eared-pheasant’s current distribution is spread across the three areas of Shaanxi, Shanxi, and Hebei-Beijing. We respectively divided the three current distribution areas. The difference between the historical distribution and the current distribution were compared in ArcGIS10.0.

### Environmental variables

The ecological and biogeographical features (habitat requirements, vegetation characteristics, environmental tolerances, and distribution sites, etc.) of the brown eared-pheasant have been well documented (Liu, Su & Ren, 1991; Pang, Ma & Liu, 2009; Zheng, 1978; Zhang, Zhang & Song, 2000; Li et al., 1990; Zhang et al., 2003). We selected 11 environmental variables that are believed to influence the distribution of the brown eared-pheasant (Table 1).

**Table 1:** List of ecogeographic variables used in Maxent model.

Environmental variables	Unit
Vegetation	
Elevation	M
Aspect	(°)
Slope	(°)
Maximum temperature	°C
Minimum temperature	°C
Annual mean temperature	°C
Annual precipitation	mm
Distance to nearest river	km
Distance to nearest road	km
Distance to nearest residential area	km

Vegetation information was collected from the Chinese vegetation-type spatial distribution map (1:1,000,000, 1980s). Elevation, slope, and aspect were obtained from a spatial distribution map of geomorphic types in China (1:1,000,000) (Aspect definition: 360 degrees divided into eight sectors, each 45 degrees (Jia, Duan & Qiao, 2007)). These data sets were provided by the Data Center for Resources and Environmental Sciences (RESDC) of the Chinese Academy of Sciences (http://www.resdc.cn). We also collected data on the proximity of rivers (China Pyatyi river map 1:100,000), roads (China road map 1:100,000), and villages and towns (China county level administrative region map 1:100,000) for each modern site in which brown eared-pheasants have been sighted. For this purpose, we used a GIS based on maps downloaded from the National Administration of Surveying, Mapping and Geoinformation, National Dynamic Atlas (http://www.webmap.cn/), which were corrected in ArcGIS10.0 (Environmental Systems Research Institute, 2011) using the Xi’an 1980. Meteorological data were obtained from fine-scaled climate data sets (WorldClim) at a spatial resolution of 2.5 arc-minutes for the period of 1950–2000 (see http://www.worldclim.org). Four climate variables were used as predictors: the annual mean temperature, the maximum temperature of the warmest month, the minimum temperature of the coldest month, and annual precipitation. Environmental factors were unified into 11 digital layers in ArcGIS10.0, spatial resolution of 5 × 5 km.

### Modeling approach

SDMs are useful tools for analyzing species–environment relationships (Marsili-Libelli, Giusti & Nocita, 2013) and predicting species distributions in biogeography, conservation biology, and climate change research (Guisan & Thuiller, 2005). SDMs include such approaches as machine learning models (e.g., MaxEnt and GARP), regression models (e.g., GAMs, GLMs, MARS, and BRT), and bioclimatic envelope models (e.g., Bioclim), all of which are now widely used (Zhang et al., 2013). Although there are concerns regarding the reliability of SDMs in forecasting the effects of habitat change related to climate (Pearson & Dawson, 2003), these models can still provide useful information when used carefully (Lobo, Jiménez-Valverde & Hortal, 2010; Acevedo et al., 2012).

Model selection was a pivotal process in choosing the most accurate predictors of the bird’s distribution (Johnson & Omland, 2004). Comparative studies have consistently shown that MaxEnt has excellent performance and outperforms many other methods (such as GARP) in estimating potential species distributions, particularly when sample sizes are small (Phillips, Anderson & Schapire, 2006). MaxEnt is a general purpose method that uses presence-only occurrence data and environmental variables in niche modeling (Phillips & Dudík, 2008), and uses a maximum-entropy approach for modeling species habitats to predict the potential geographic distribution of a species (Wang, 2014). When MaxEnt is used to model a geographical distribution over a certain period of time, the division of time is of great importance in the modeling (Zhang et al., 2013). We divided 25–1947 CE as the historical period base on historical records, and 1948–2000 as the occurrence period. The historical data were not used in Maxent.

The MaxEnt model was run with 15 replicates for balance error. The model was calibrated for each replicate, using a random sample of 75% of the modern distribution data for model training (n = 34). We evaluated this against the remaining 25% for testing (n = 11), and randomly generated background points (10,000) within the local range, with a maximum 5,000 times iterations.

Omission and the area under the receiver operating characteristic curve (AUC) score are commonly used to measure the predictive performance of a model (Pearce & Ferrier, 2000). The omission range is from 0 to 1, where lower omission values are indicative of higher prediction accuracies (Kang, 2010).

Although some studies criticized the AUC for evaluating model performance (Lobo, Jiménez-Valverde & Real, 2008; Peterson, Papeş & Soberón, 2008), AUC still widely used in the testing of presence-only species distribution modeling due to the fairly objective evaluation results (Raes & ter Steege, 2007). Usually AUC ranges is 1.0–0.5, AUC values higher than 0.7 are considered to give useful predictive results (Fielding & Bell, 1997). We used two statistical analyses (OR and AUC) to evaluate the model’s performance.

In order to classify the suitable habitat, we need binary maps obtained from continuous probability models by setting a threshold value above potentially suitable area. A good rule for determining an appropriate threshold would depend on the predicted values assigned to the training localities, the number of training localities and the context in which the prediction is to be used (Phillips, Anderson & Schapire, 2006). We used the 10% TPLT (10% Training Presence Logistic Threshold) value as threshold to ensure a low omission rate. The 10% TPLT can be easily argued on ecological grounds since it includes 90% sites at least as suitable as those where the species has been recorded (Pearson et al., 2007). The desired predicted area can be obtained from Maxent model by a perfect threshold. The thresholds and resulting predicted areas were chosen to facilitate the statistical analysis of results. We calculated potential distribution area as determined by 10% TPLT in ArcGIS10.0.

We used the software MaxEnt to build distribution model for predicting the geographical distribution of brown eared-pheasant on the basis of current distribution records and 11 environmental factors. Then we compared the predicted potential distribution with the historical distribution in ArcGIS10.0 to verify the historical distribution decrease.

We used the Random Forests measure from the R3.0.2 software platform to analyze the importance of the 11 environmental variables and their effect on species distribution. Spatial information extraction tools were used to extract habitat factors from 45 current distribution points within the 11 digital layers. The Mean Decrease Accuracy (MDA) and Mean Decrease Gini (MDGini) index were used to determine the importance of factors using RF, with larger MDA values indicating greater importance (Jin et al., 2014).

### Forest cover

The historical distribution of the brown eared-pheasant covered all of the Shanxi (Liu, Su & Ren, 1991; Zhang, 2015), and Shanxi was the most concentrated distribution of this species, historically and recently. Shanxi was selected as the main study area.

Vegetation may influence species ranges (Harris & Pimm, 2008) and are important for explaining species distributions (Newbold et al., 2009). Vegetation determines several habitat factors and can be used as an important habitat index for terrestrial animals (Scott et al., 1993). The brown eared-pheasant occurs mainly in coniferous and mixed coniferous-broadleaf forests. The species is a plant-based diet, eating some insects in the breeding season (Liu, Su & Ren, 1991). The ecological and biogeographical features (habitat requirements, vegetation characteristics, environmental tolerances, and distribution sites, etc.) of the brown eared-pheasant have been well documented (International Union for Conservation of Nature, 2015; Pang, Ma & Liu, 2009; Zhang, Zhang & Song, 2000; Zheng, 1978; Li et al., 1990; Zhang et al., 2003). The food availability is the statistically significant factor for brown eared-pheasant (Liu, Su & Ren, 1991; Zhang, 2015). The forest cover had changed substantially which lead to the loss of food resources according to botanical and ecological data (Qu & Mi, 2009a). The relationship between the distribution of brown eared-pheasant and forest coverage was the key for understanding the ecological and biogeographic characteristics of species distribution decline.

Consequently, we built a correlative relationship between the presence/absence of brown eared-pheasant and forest coverage in each historical period throughout Shanxi to explore forest cover effects on this species. We defined the last record time of historical distribution point as the cut-off point for occurrence time, the occurrence before the demarcation time as a presence which from the last record time to 25 CE, and from the demarcation time to the 2,000 year as an absence. The presence/absence value of the brown eared-pheasant was defined as either 1 or 0, where 1 indicates presence and 0 indicates absence.

Definition of presence/absence of brown eared-pheasants as:

$$25\;CE{{} \over {presence(1)}}\overbrace \bullet ^{demarcation}{{} \over {absence(0)}}2000\;CE$$

The data used to describe forest cover in each time period throughout Shanxi (within 25–2000 CE) were collected from monographs (see Qu & Mi, 2009a; Ma, 2001; Qu, Liu & Han, 1999; Qu, Di & Zhao, 2004) and papers (see Cui, 2008; Sang, 2005). This correlative relationship was analyzed using SPSS19.0.

## Results

### Distribution of the brown eared-pheasant

**Figure 1:**
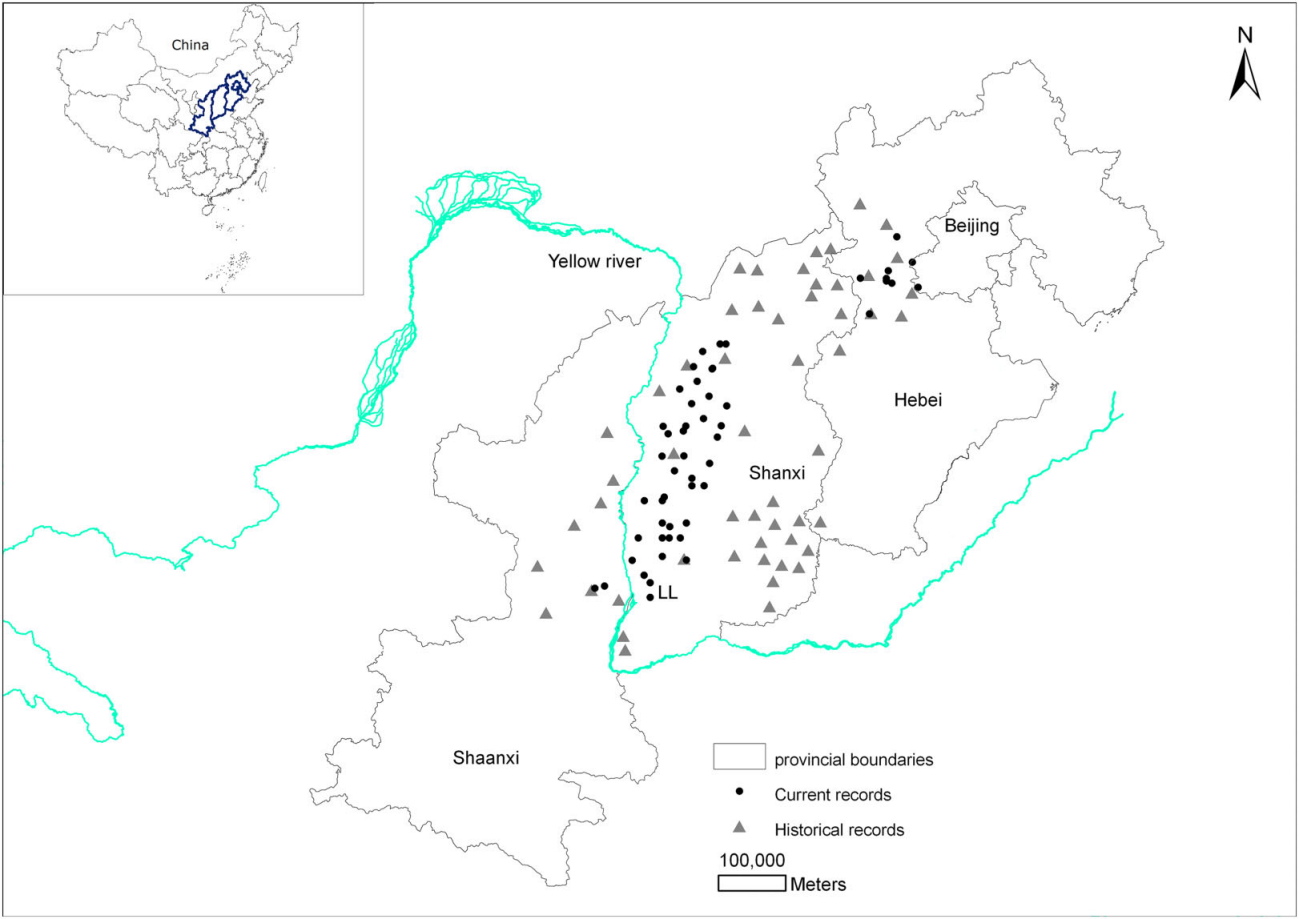
Changes in the distribution records of the brown eared-pheasant in China.

There are historical records of brown eared-pheasants occurring in 13 Chinese provinces (Shaanxi, Shanxi, Hebei, Beijing, Henan, Anhui, Hubei, Sichuan, Fujian, Guangdong, Liaoning, Heilongjiang, and Gansu). 10 provinces records were excluded and we finally determined that the brown eared-pheasant was historically widely distributed in China, including Shanxi, the east and center of Shaanxi, and the west and center of Hebei. According to the Site Record Database for Chinese Galliformes, the current distribution area of this species includes Huanglongshan in Shaanxi, the Luliang Mountains in west Shanxi, the XiaoWutai Mountains in Hebei, and parts of the Baihua Mountains in Beijing (Fig. 1).

The distribution has decreased considerably, with most of the original distribution area being lost over the past 2,000 years. The most serious reduction in the historical distribution area has occurred in Shanxi.

### MaxEnt model results

All of the training omission error values were < 0.09, and all of the test omission error values were < 0.33. These values indicate that the MaxEnt model has very high prediction accuracy. The training AUC of 0.9575, the test AUC of 0.8985, and AUC standard deviation of 0.0406 are indicative of good model prediction performance (Table 2).

**Table 2:** The omission indexes and the AUC values of prediction by MaxEnt model. AUC standard deviation reflect the discrete extent of the data set.

Indexes	Vaule
Minimum training presence training omission	0
Minimum training presence test omission	0.1185
10 percentile training presence training omission	0.0741
10 percentile training presence test omission	0.3037
Equal training sensitivity and specificity training omission	0.0963
Equal training sensitivity and specificity test omission	0.3333
Maximum training sensitivity plus specificity training omission	0.0691
Maximum training sensitivity plus specificity test omission	0.3259
Training AUC	0.9575
Testing AUC	0.8985
AUC standard deviation	0.0406

**Figure 2:**
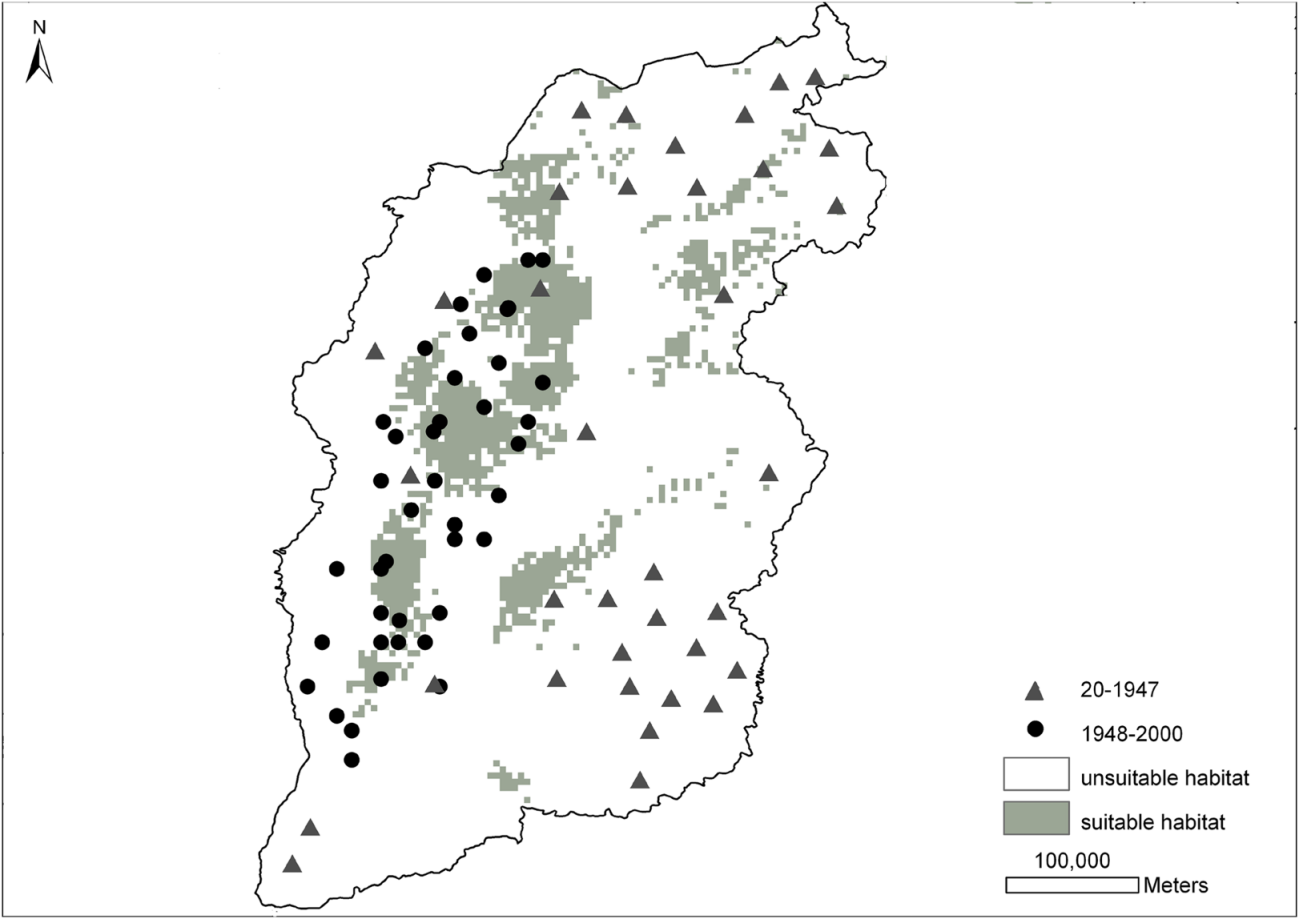
Distribution probability map of brown eared-pheasant was predicted by the MaxEnt model. The gray triangles indicate the historical localities of brown eared-pheasant (25–1947 CE). The black points represent the current distribution (1948–2000 CE). Gray areas indicate potentially suitable distributions, white areas indicate unsuitable habitat. Western Shanxi is the concentrated distribution area of the brown-eared pheasant, and the predicted range overlapped with the current distribution. Northern, northeastern, southeastern, and southwestern regions were all within the historical distribution range.

Figure 2 shows the prediction probability map for the brown eared-pheasant distribution. The historical distribution of this species covered most of the Shanxi province, whereas its modern distribution has been mainly restricted to the Luliang Mountains. The grey area which indicate a high presence probability, have mainly been concentrated in the panhandle of the western, and small parts in northeast and midland of Shanxi. This predicted range mostly overlapped with the current distribution records. The white area indicates a low presence probability where included the northern, northeastern, eastern, southeastern, and southwestern areas. These areas were included in this species’ historical distribution range. However, there have not been any new reports of this species in these locations. The prediction results of the potential distribution of brown eared-pheasant support observations of a reduction in the historical distribution of this species, and only western Shanxi have consecutive suitable distribution areas.

### Decrease of forest cover in the Shanxi province

The Random Forests results (Table 3) showed that vegetation (MDA = 17.34, MDGini = 5.38) had the largest MDA and MDGini index values than the other 10 factors; elevation, slope, annual mean temperature, annual precipitation, distance to nearest residential area had intermediate index values, while the other five factors had low index values. Vegetation were therefore considered to be the main factors affecting the distribution of this species.

**Table 3:** Importance-evaluation of environmental factors by RF.

Environmental variables	MDA	MDGini
Vegetation	17.3407791	5.3800819
Elevation	13.247365	5.1929068
Aspect	1.7693794	0.8643999
Slope	5.6271948	2.4516629
Maximum temperature	0.7572997	0.5847518
Minimum temperature	1.0193455	0.6115456
Annual mean temperature	5.0940214	1.2410759
Annual precipitation	9.2005122	2.4756012
Distance to nearest river	1.7516348	1.0119277
Distance to nearest road	−0.2775467	1.4151484
Distance to nearest residential area	6.5675466	2.1830715

**Figure 3:**
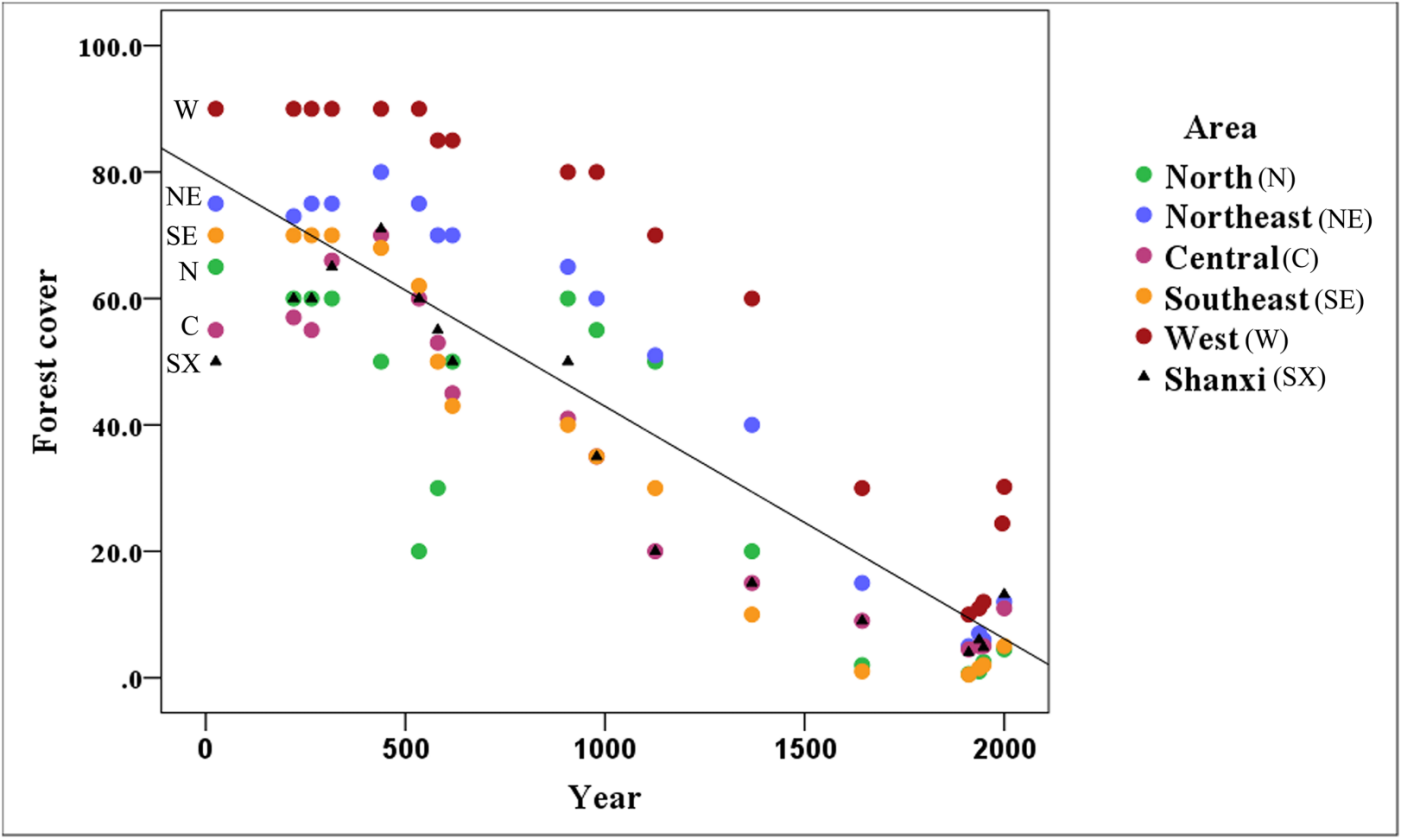
Variation of forest cover throughout the Shanxi province. The amount of forest coverage in the northern (Datong area), northeastern (Wutai area), central (Taiyuan area), southeastern (Changzhi area), and western (Luliang area) of Shanxi has declined annually. The north and southeast areas have suffered the most serious declines; however, there is still a relatively high amount of forest coverage in the western part of the Shanxi province.

Figure 3 shows that the area covered by forests has decreased markedly in Shanxi (R^2^ = 0.747), with forest coverage declining continuously from the reported 50% in 25 CE to 4.8% in 1948 followed by a recovery to 13.2% in 2,000 based on historical records.

The forest coverage in the northern, northeastern, central, southeastern and western regions of Shanxi has declined. In all of these regions, forest cover exceeded 10% before the Ming Dynasty (1368 CE), but the northern, northeastern, central, and southeastern regions have declined to 0.6, 5.0, 4.5, and 0.5% forest cover, respectively, over the late Qing Dynasty (1911 CE), followed by a recovery to about 5% by 2,000. In addition to the general decline in forested areas, there was a notable sudden decline, likely associated with the Western Jin Dynasty (316 CE), and a decline to 20% in the later period of the Northern Wei Dynasty (534 CE) in northern Shanxi, followed by a recovery back to 60% until the end of the Tang Dynasty (907 CE). The most dramatic changes occurred in northern and southeastern Shanxi, which experienced some of the most serious declines. The forest has remained relatively intact in western Shanxi. The forest cover was maintained at about 10% during the late Qing Dynasty (1911 CE) in the Luliang Mountains, which remains the primary distribution area for this species.

**Figure 4:**
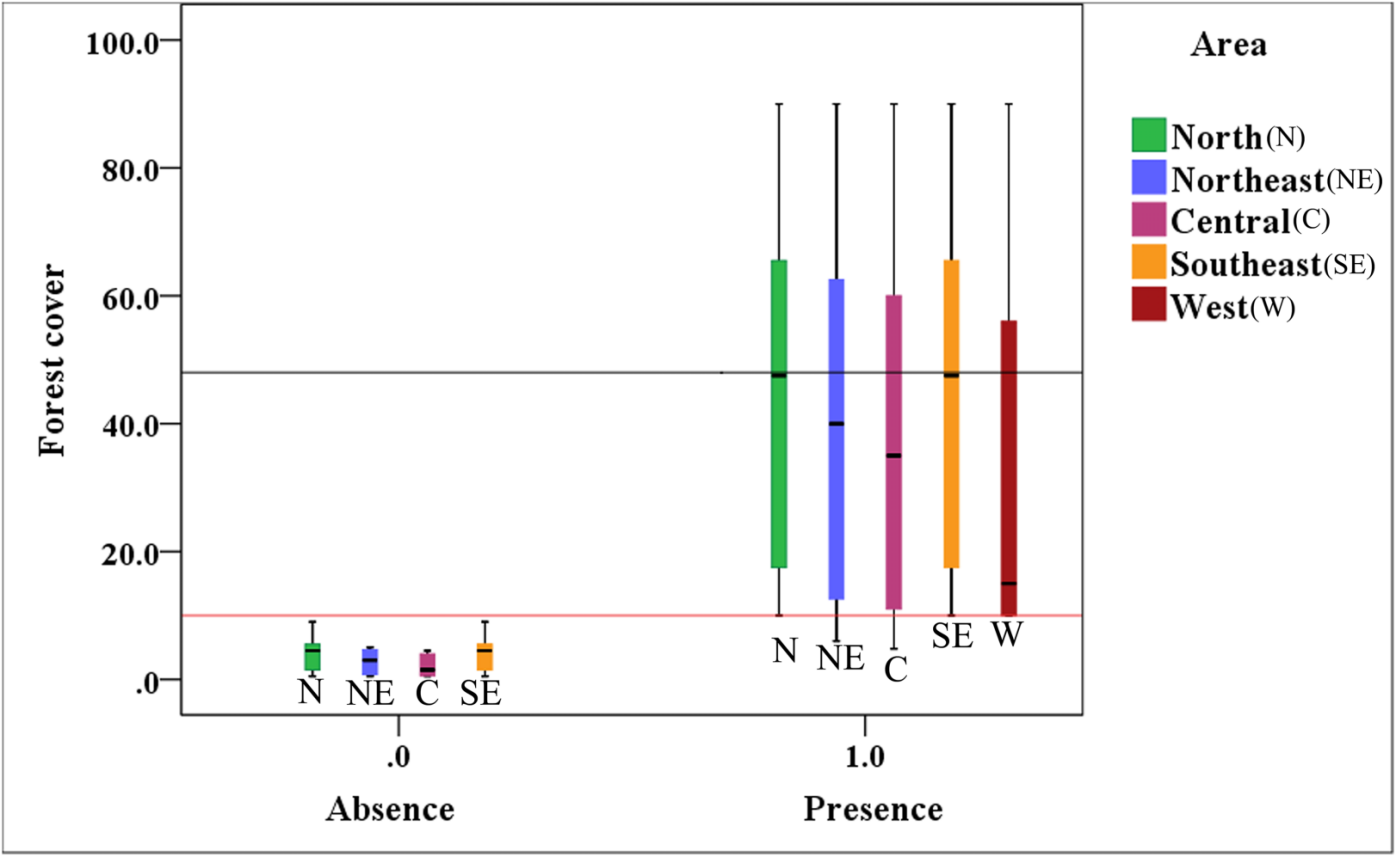
Correlative relationship between the presence/absence value of brown eared-pheasant and forest coverage in the Shanxi province. Brown eared-pheasant appeared in all districts of Shanxi when the forest coverage was greater than 10%. There has been local extinction in northern, northeastern, central, and southeastern regions when the forest cover rates were less than 10%. The western areas of the province have always occupied the main distribution area of this species because of forest coverage has remained above 10%.

Figure 4 indicates that the presence value of the brown eared-pheasant was one in all of the districts of the Shanxi province when forest coverage of the historical distribution was over than 10%, and the max-median of the forest cover was 48%. The absence value was 0 in northern, northeastern, central, and southeastern Shanxi when the forest coverage was less than 10%. This species is still present in the western areas where forest coverage rates are over 10%. It is likely that there will be a regional extinction when less than 10% of the forest coverage area remains. Thus, we speculate that 10% forest cover is probably the minimum threshold for maintaining the brown eared-pheasant distribution, and 48% forest cover is likely to guarantee stable in the distribution of the brown eared-pheasant.

## Discussion

### The historical distribution range of the brown eared-pheasant

The long-term historical records are very important for understanding the overall distribution of the species, but some records are still restricted by practical barriers, including data accessibility, spatially and temporally variable and non-standardized sampling (Davies, Colombo & Hanley, 2014). Furthermore, we doubt the accuracy of some of the records because the climate of the provinces was inappropriate for brown eared-pheasant and for some locations there was only a single isolated record may be associated with data shortage. Thus we deleted these dubious records for acquiring accurate results.

The forest is the divide between the warm temperate deciduous broad-leaved forest and north subtropical evergreen deciduous and broad-leaved mixed forest zones of the Qinling Mountains (Luo et al., 2011). The climate and vegetation conditions of the northern and southern slopes of the Qinling Mountains are different, and representing a transitional zone in the bird’s distribution (Zheng, 1973). From the historical vegetation and climate data obtained for south China, we found that the tropical and subtropical climate conditions were stable, with constant luxuriant tropical rain forest and subtropical monsoon forest flora since the Mesozoic (Institute of Botany in Guangdong, 1976; Zhang, 1993). These areas did not provide a suitable habitat for the brown eared-pheasant, and the species should therefore not be present south of the Qinling Mountains. This was in accordance with the distribution of *Crossoptilon* in a description of China’s zoogeographical regions (Zheng & Zhang, 1956).

We obtained a relatively complete distribution records for the brown eared-pheasant by consulting a large number of historical data. Consequently, we considered that the historical distribution range of the brown eared-pheasant included east and central Shaanxi, Shanxi, and west and central in Hebei. This historical distribution range overlapped and encompasses the current distribution.

The current distribution exhibits severe fragmentation (Liu, Su & Ren, 1991), with three main areas: a west population in the Huanglongshan in Shaanxi, a midland population in the Luliang Mountains in Shanxi, and an east population in Hebei-Beijing (Zhang, Zhang & Song, 2000). The current distribution is a discontinuous island (Wang, Chen & Lai, 1985; Li & Wei, 1993).

Altogether the brown eared-pheasant geographic distribution presents a sharp decline. We concluded that most of the historical area was no longer appropriate for this species; Shanxi province losing the largest amount of distribution area. The historical data have considerable potential to contribute to ecological baselines for informing conservationists (Yang et al., 2016). The shrinking distribution area might be stronger than our result in consideration of the historical data may be missed because of incomplete records, such as surveyors fail to detect existent individuals or miss the remote areas in the local gazetteers.

### MaxEnt model verification

Figure 2 indicates that the MaxEnt model predicted the distribution probability of the brown eared-pheasant mainly in west area Shanxi. The distribution records of this species had been really reduced over the 2,000 years. MaxEnt just generates hypotheses about a species distribution, rather than modeling the actual suitability of potential habitats (Hortal, Lobo & Jiménez-Valverde, 2012). The north, northeast, east, southeast, southwest and central regions of Shanxi all had historical reports of brown eared-pheasant before the Qing Dynasty (1368 CE), but there have been no current reports from these areas. The forest cover declined in these areas. It is likely that these areas provided suitable habitats for the brown eared-pheasant in historical times. Nevertheless, the predicted result shows that the western regions of Shanxi were suitable for the brown eared-pheasant. The current distribution area is concentrated in the Luliang Mountains in western Shanxi.

Factors other than vegetation (e.g., humidity, land use, or extreme climatic conditions) may influence the predicted results. Furthermore, geographic barriers, climatic history, the evolutionary history of the species (Hortal, Lobo & Jiménez-Valverde, 2012), and its dispersal ability (Peterson et al., 2002) all are related to species distributions, resulting in predictions of species distributions that may differ from their true distributions. Overall, the MaxEnt simulation result confirmed that the western Shanxi is the main distribution area for this species. The results corresponds largely to the current distribution, and the historical distribution of this species is shown as having shrunk in accordance with the historical facts.

### The relationship between forest cover and the distribution

Vegetation determines several habitat factors and can be used as an important habitat index for terrestrial animals (Scott et al., 1993). The study showed the continued reduction in the distribution range of the brown eared-pheasant had been accompanied by the loss of forest cover in Shanxi. The vegetation cover has changed substantially in Shanxi (Qu & Mi, 2009a), we consider that vegetation change can well explain the loss of range area.

Shanxi province is the main distribution area of the brown eared-pheasant. Figure 3 shows that Shanxi had lush forests in the past, with 10–70% forest coverage before the Ming Dynasty (1368 CE), and brown eared-pheasant were abundant. However forest coverage gradually diminished from 50% in 25 CE to 4.8% in 1948. The forests were almost completely destroyed in the Ming and Qing Dynasties (1368–1911 CE) (Zhang, Zhang & Song, 2000). When the forest cover rate of Shanxi dropped to 4% at the end of the Qing Dynasty (1911 CE), no more records of brown eared-pheasant were reported in many area (Liu, Su & Ren, 1991; Zhang, 2015; Harris & Pimm, 2008; Qu & Mi, 2009b).

The brown eared-pheasant probably became locally extinct because of forest destruction in many parts of its former range. For example, the north, northeast, central, and southeast areas of Shanxi were important historical distribution areas of this species. Before the Ming Dynasty (1368 CE), the historical distribution was broad (Fig. 1), and forest coverage in these regions exceeded 10% (Fig. 3). However, no new records of brown eared-pheasant from these areas were subsequently reported. The forests of west and northwest Shanxi were better preserved than those of other regions (Qu & Mi, 2009a). The Luliang Mountains represents the most important current distribution area of brown eared-pheasant in West Shanxi.

The northeast, east, central, southeast, and southern areas were the locations of major battlefields in the history of Shanxi (Qu & Mi, 2009a), and forests were seriously damaged due to deforestation and wars in these locations (Qu & Mi, 2009a). The suitable habitat for the brown eared-pheasant had been drastically reduced as a result of forest destruction associated with historical wars and post-war reconstruction. The forest was very seriously destroyed in the north (Datong area) and southeast (Changzhi area), with forest cover extremely low at the end of the Qing Dynasty (1911 CE) (Qu, Di & Zhao, 2004). In the central basin (Taiyuan), forest coverage was maximal at 70% in 439 CE, then deforestation and tillage resulted in forest loss subsequent to wars, and the brown eared-pheasant disappeared in Taiyuan during the Qing Dynasty (1840 CE) (Qu, Liu & Han, 1999). There were many temples in the northeast (Wutai Mountains), where forests survived the wars for a short time, but these were eventually destroyed (Qu & Mi, 2009b). The north, northeast, central, and southeast regions all represent historical distribution areas of the brown eared-pheasant. The bird had not disappeared completely in the southeast (Changzhi), at least in the early Qing Dynasty (1644 CE) (Liu, Su & Ren, 1991), but it had become locally extinct in the northeast and central areas in the Late Qing Dynasty (1911 CE) (Qu, Liu & Han, 1999; Qu & Mi, 2009b). Due to the long-term impact of human activity on forests in Shanxi, especially the large-scale deforestation and destruction in the Ming and Qing Dynasties (1368–1911 CE), a reduction of forest area ensued (He & He, 1990). The species could not be found at these historical locations following the disappearance of its required habitat. The forest survived in the west and northwest in Shanxi and in the western Luliang Mountains (Qu & Mi, 2009a), where the brown eared-pheasant can still be found today.

Forest coverage increased to 13.2% in 2,000 in Shanxi, where four national nature reserves were established for the conservation of the species and its habitats: Luyashan Nature Reserve, Pangquanguo Nature Reserve, Wulushan Nature Reserve, and Heichashan Nature Reserve. The populations within these protected areas appear to be stable (International Union for Conservation of Nature, 2015; Zhang, Zhang & Song, 2000).

The brown eared-pheasant requires a suitable forest habitat (Liu, Su & Ren, 1991; Zhang et al., 2003), with populations declining and local extinction occurring due to habitat loss. The current distribution area is smaller than the historical distribution. Most of this species` original distribution area has disappeared in the past 2,000 years at the same time as the forest cover declined sharply. This indicates that the loss of forest resources was synchronous with the reduction of the distribution range of this species, and forest cover decline was closely related to the contraction in its distribution range.

The brown eared-pheasant widely exist in Shanxi when forest cover was over than 10%, the max-median of the forest cover was 48%. The species absent in the north, northeast, central, and southeast areas of Shanxi followed forest cover lower than 10%. We consider the lowest threshold of forest cover for ensuring this species` survival to be at 10%. Therefore, we suggest that to guarantee stable growth in the population and distribution of brown eared-pheasant, forest coverage should not be less than 48% in the natural reserves where the brown eared-pheasant is currently distributed.

A clear consistency was found between the loss of forest cover and a reduction in the distribution area of the brown eared-pheasant. The brown eared-pheasant is an endemic mountain forest bird that cannot exist without its forest habitat. Hence, the distribution of the brown eared-pheasant is limited by forest cover. We expect the population and distribution of this species to remain stable growth when forest cover is greater than 48% in the conservation areas. Further studies are needed to deepen our understanding of the relationship between regional forest cover and species distribution.

## Supplemental Information

10.7717/peerj.2556/supp-1 Supplemental Information 1 The current distribution data of the brown eared-pheasant used in the Maxent model.

10.7717/peerj.2556/supp-2 Supplemental Information 2 Sources of information mentioned in the text for the ancient records of Brown eared pheasant (i.e. 51 ancient books, 149 references, 7 monographs).

## Additional Information and Declarations

### Data Deposition

The following information was supplied regarding data availability: The raw data has been supplied as Supplemental Dataset Files.
