# Supplementary material for: Determining the distribution loss of brown eared-pheasant (Crossoptilon mantchuricum) using historical data and potential distribution estimates
Source: PeerJ. 2016 Oct 19;4:e2556. doi: 10.7717/peerj.2556 (PMC5075714; doi:10.7717/peerj.2556)
Supplement: Supplemental Information 2 [file peerj-04-2556-s002.doc]

**Supplementary Data**

**S1 Table. Sources of information mentioned in the text for the ancient records of Brown eared pheasant (i.e. 51 ancient books, 149 references, 7 monographs).**

| **Category** | **Data source** |
| --- | --- |
| Ancient books | Beijing Forestry University Library, National Library of China, National Digital Library of China |
| References | Beijing Forestry University Digital Library, National Digital Library of China, China Knowledge Resource Integrated Database |
| Monographs | Beijing Forestry University Library, National Library of China |
